# Selenium Cycling Across Soil-Plant-Atmosphere Interfaces: A Critical Review

**DOI:** 10.3390/nu7064199

**Published:** 2015-05-29

**Authors:** Lenny H.E. Winkel, Bas Vriens, Gerrad D. Jones, Leila S. Schneider, Elizabeth Pilon-Smits, Gary S. Bañuelos

**Affiliations:** 1 Swiss Federal Institute of Technology (ETH), Institute of Biogeochemistry and Pollutant Dynamics, ETH Zurich, CH-8092 Zurich, Switzerland; E-Mails: bas.vriens@eawag.ch (B.V.); leilasc@student.ethz.ch (L.S.S.); 2 Eawag: Swiss Federal Institute of Aquatic Science and Technology, Ueberlandstrasse 133, P.O. Box 611, CH-8600 Duebendorf, Switzerland; E-Mail: lenny.winkel@eawag.ch (L.H.E.W.); bas.vriens@eawag.ch (B.V.); gerrad.jones@eawag.ch; 3 Colorado State University, Biology Department, Fort Collins, CO 80523, USA; E-Mail: epsmits@lamar.colostate.edu; 4 USDA, Agricultural Research Service, San Joaquin Valley Agricultural Center, 9611 South Riverbend Avenue, Parlier, CA 93648, USA; E-Mail: gary.banuelos@ars.usda.gov

**Keywords:** selenium, environment, plants, soil, atmosphere, speciation, biomethylation, biofortification, hyperaccumulation

## Abstract

Selenium (Se) is an essential element for humans and animals, which occurs ubiquitously in the environment. It is present in trace amounts in both organic and inorganic forms in marine and freshwater systems, soils, biomass and in the atmosphere. Low Se levels in certain terrestrial environments have resulted in Se deficiency in humans, while elevated Se levels in waters and soils can be toxic and result in the death of aquatic wildlife and other animals. Human dietary Se intake is largely governed by Se concentrations in plants, which are controlled by root uptake of Se as a function of soil Se concentrations, speciation and bioavailability. In addition, plants and microorganisms can biomethylate Se, which can result in a loss of Se to the atmosphere. The mobilization of Se across soil-plant-atmosphere interfaces is thus of crucial importance for human Se status. This review gives an overview of current knowledge on Se cycling with a specific focus on soil-plant-atmosphere interfaces. Sources, speciation and mobility of Se in soils and plants will be discussed as well as Se hyperaccumulation by plants, biofortification and biomethylation. Future research on Se cycling in the environment is essential to minimize the adverse health effects associated with unsafe environmental Se levels.

## 1. Introduction

The trace element selenium (Se) is essential for human and animal health, while no Se requirement has been shown for higher plants [1,2,3,4]. The role of Se in physiology is mainly derived from its presence in the Se-containing amino acids selenomethionine (SeMet) and selenocysteine (SeCys). The latter has been termed the 21st amino acid since it is needed in a small set of selenoproteins that serve redox functions [5]. Selenoproteins include glutathione peroxidases and thioredoxin reductases [6,7,8], which have a variety of functions including protection from oxidative damage, regulation of intracellular redox state and thyroid hormone metabolism [6]. Adequate Se intake levels have a relatively narrow range between deficiency and toxicity: current estimates indicate that an intake of Se of 30 μg·day^−1^ is inadequate for humans, while intakes exceeding 900 μg·day^−1^ are potentially harmful [9]. Dietary Se intake levels mainly depend on the total concentration and bioavailability of Se in food sources (crops, fish and animal products), which depend on the multiple factors such as Se content of soils, irrigation water and animal feed. Selenium content has been found to vary considerably across food sources and geographical origin, and Se deficiency is more widespread than Se excess, both in agricultural and natural areas [10,11].

Apart from the total Se content of food sources, the chemical form, *i.e.*, speciation, is important, as it affects the bioavailability and nutritional value of Se. The various chemical forms of Se exhibit highly different properties with regard to sorption, bioavailability, mobility and toxicity [12,13,14,15]. To prevent Se toxicity and to provide adequate Se in the human diet, it is of great importance to obtain insight into the processes that govern the distribution and speciation of Se in agricultural soils and plants. This review is aimed at summarizing the current knowledge of Se cycling across the soil-plant-atmosphere interfaces. These interfaces are of major importance as they control the local-scale mobility of Se in agricultural and natural systems and potentially also play an important role in the global Se distribution. Particular focal points are plant uptake and biomethylation since both are key distribution processes that highly contribute to the mobility of Se in the environment. In addition to sources and sinks of Se in the environment as well as the biotic and abiotic transformation processes of Se in soils and plants, incl. biofortification and Se hyperaccumulation, this review also identifies knowledge gaps and missing links in/between the different areas of research.

## 2. Selenium in Crops

For organisms that mainly consume a plant-based diet, health problems related to Se deficiency may arise due to selenium’s variable distribution in the soil and its moderate uptake by crops. Selenium toxicity is a problem in areas with seleniferous soils, including parts of China, India and the USA, while Se deficiency is a problem in areas where soil Se is particularly low, such as other parts of China, the USA as well as Australia, New Zealand and Finland [16]. Since low concentrations of plant-available forms of Se in soil can decrease the dietary intake of Se, it is very important to improve the bioavailability of Se and increase the Se content in plant food-products in Se-deficient areas, e.g., China and Finland. Therefore, management strategies aimed at minimizing Se deficiency and associated diet-related disturbances should be focused on establishing a connection between agricultural food-products and Se content and bioavailability in soil.

### 2.1. Biofortification

One of the most promising approaches to combat a low transfer of Se from soil into the food chain involves a concept called Se biofortification [17]. This agronomic-based strategy can be utilized by growers to produce Se-enriched food products that may help reduce dietary deficiencies of Se occurring throughout susceptible regions of the world [18]. Increasing the dietary intake of Se, as well as the bioavailability and accumulation of Se to food crops, may be accomplished by amending soils with Se, adjusting cropping systems, selecting Se accumulating cultivars, and using modern genetic engineering technologies.

#### 2.1.1. Speciation of Se in Crops

The major determinant of Se bioavailability in Se-enriched food sources is the Se speciation [19]. Although Se may also be present in inorganic forms (e.g., selenite [Se(IV)], selenate [Se(VI)]) in crops [6], the predominant form of Se extracted with enzymatic hydrolysis is generally organic: selenomethionine (SeMet) (e.g., in biofortified wheat [20]), methylselenocysteine (MeSeCys) (e.g., in carrots and onions [21,22] and broccoli [23]) and γ-glutamyl-Se-selenomethyl-selenocysteine (γ-Glu-MeSeCys) (e.g., in carrots and onions [21,22]). More research is needed to determine how application doses and chemical forms of applied Se to the soil or foliar surfaces affect Se speciation and the proportion of organic Se species accumulated in edible plant tissues. The organic forms of Se available in biofortified products are the preferred choice for long-term population-wide Se supplementation strategies [24] as they may have additional health value under Se-deficient conditions [25]. Hence, to elucidate the resulting forms and quantities of Se-amino acids in food products resulting from a biofortification practice more research on Se speciation is needed.

#### 2.1.2. Application of Se to Soils

The strategy of Se biofortification of food crops is practiced in Se-deficient regions by adding inorganic Se-containing fertilizers to soils, e.g., in Finland [26,27], UK [28,29], and New Zealand [30], or by adding organic sources of Se to soils, e.g., in the USA [23]. With either source of Se, the efficacy for biofortification of crops with Se strongly depends on the physical, chemical and biological properties of the soil (as discussed in Section 4.2) [31]. Although Se-amended inorganic fertilizers can be beneficial for producing Se-biofortified food and animal feed products [18], disadvantages include low Se recovery rates in edible portions of crops [28] or excessive Se accumulation in the soil with long-term application of Se fertilizer that might become toxic for nearby ecosystems [32]. An effective method of biofortifying food crops with Se is foliar application. Selenite and selenate have been shown to be more bioavailable to plants when applied directly to leaf surfaces opposed to soils [21]. Therefore, foliar application of Se may be a more efficient method of biofortification, especially considering that soils can be effective sinks for Se. Importantly, the direct foliar exposure route ensures a high efficiency of Se assimilation by the plant since it does not depend on root-to-shoot translocation. The rate of biotransformation from absorbed inorganic Se to different organic forms of Se in a plant is species specific and depends on specific biochemical pathways [33].

### 2.2. Genetic Engineering

Genetic engineering for the promotion of higher Se levels in plant has been reviewed earlier by others [34,35] and applied to *Brassica* crops in field conditions [36,37]. In addition to genetic manipulation, the interspecific and intraspecific variation in Se accumulation within plant taxa may be exploited for biofortifying food crops [4]. The selection of existing cultivars for their higher accumulation of Se, as well as for the forms of organic Se distributed in the edible parts of a given crop can have an immediate impact on daily dietary Se intake, especially if such cultivars can be used in crop breeding programs. Thus, more effective biofortification could be achieved if plant breeders, molecular biologists, and agronomists collaborate to better understand the limiting factors of Se uptake, accumulation and biotransformation in plants.

#### Using Naturally-Enriched Se Materials

Despite the widespread occurrence of Se deficiency, some soils, mineral deposits and waters are naturally rich in Se, and exploitation of those seleniferous resources in agricultural practice can lead to an increased Se accumulation in plant tissues. Bañuelos (2002) [38] has observed the increased accumulation of Se in broccoli and canola irrigated with waters containing naturally high levels of Se. In addition, unpublished work by Bañuelos and Freeman (2015) [39] demonstrated that Se-enriched vegetables, e.g., Swiss chard, red and green cabbage, accumulated Se ranging from 3 to 9 mg Se kg^−1^ (dry weight), when grown in soils containing high levels of naturally occurring Se. Hence, using soils naturally rich in Se for growing foods can be a viable and low-cost agronomic strategy for natural biofortification.

## 3. Sources and Sinks of Soil Selenium

**Figure 1:**
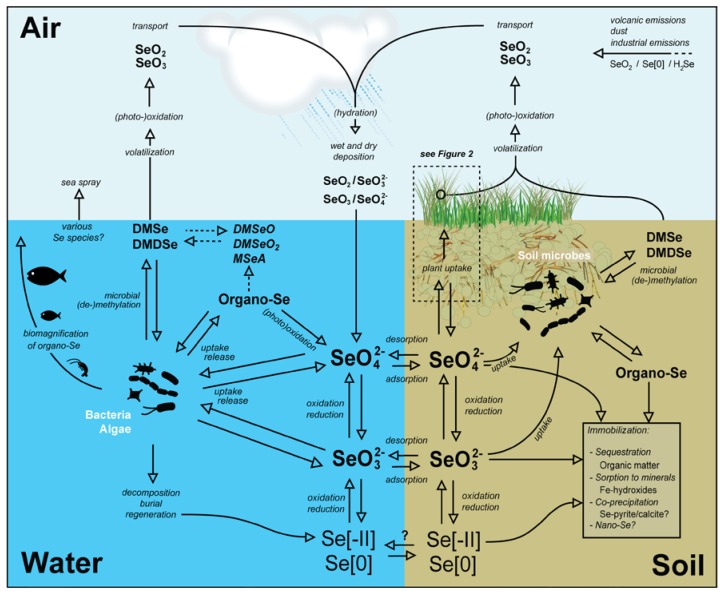
Overview of Se species, pathways and transformations in soil, water, atmosphere and their interfaces. Abiotic and biotic fluxes and transformations are indicated in italics at the corresponding arrows. Potential immobilization processes in soils are listed in the frame-inset.

In general, Se in soils can originate from both local and regional sources. Local sources include rocks (geogenic sources) from which Se can be mobilized via weathering and leaching in addition to anthropogenic sources such as Se-fertilizers. Sources that add Se to soils over medium- to long-range distances include wet and dry atmospheric deposition derived from both anthropogenic (fossil fuel burning, metal smelting, ship emissions) and natural sources (biomethylation, volcanic activity). A number of reviews have been published on atmospheric [40,41] and geogenic [13,42] sources of Se to soils (see Table 1). Figure 1 gives a schematic overview of the most important Se species in soils, water, and atmosphere and indicates the main biogeochemical pathways across the interfaces.

**Table 1:** Main reviews on various aspects of Se in the environment. Reviews on the role of Se in human and animal health, food and advances in analytical methods are not included.

Subject/Scope	Year	Reference
Overview of analytical methods, bioaccumulation, and effects of Se in agricultural environments.	1984	[43]
Overview of the state of knowledge of Se occurrence, effects, uptake, *etc.* in agricultural environments.	1989	[44]
Comparison between Se and S biogeochemical atmospheric cycling and discussion of atmospheric Se fluxes.	1990	[45]
Selenium speciation, partitioning, and volatilization in wetlands.	1993	[46]
Occurrence of Se in natural and environmental waters, incl. a review of analytical methods and speciation.	1997	[47]
Physiology and biochemistry of Se accumulator and non-Se accumulator plants, incl. Se uptake, assimilation, incorporation into proteins and volatilization.	2000	[34]
Review of solubility and acid/base equilibration constants for various Se species.	2001	[48]
Gives an overview of biomethylation of Se and tellurium (Te) by Microorganisms and plants, incl. a review of analytical techniques and proposed biomethylation mechanisms.	2003	[49]
A review of measurement techniques to investigate Se isotope fractionation and current understanding of Se isotope dynamics.	2004	[50]
Reviews Se impacts on aquatic ecosystems and food chains. The interaction of Se with other elements and effects of Se on survival and growth of fish are also discussed.	2004	[12]
Reviews studies on Se accumulation in sediments and waters due to agricultural drainage as well as effects of Se toxicity on wildlife.	2004	[51]
Review of Se concentrations and speciation in coal and of genetic types of Se-accumulations in coal.	2006	[52]
Major aspects of atmospheric Se and its natural and anthropogenic sources.	2007	[40]
Mechanistic evaluation of processes governing Se cycling and bioavailability.	2009	[13]
Covers many facets of biofortification.	2009	[17]
Covers biofortification of agricultural crops with different macro- and micronutrients. Reviews aspects of soil, physiology, agronomic, and genetic approaches to biofortify food crops with different mineral elements.	2009	[53]
Reviews key developments in the understanding of Se in higher plants and advances in genetic engineering of Se metabolism.	2009	[4]
Reviews transgenic approaches for enhancement of plant Se accumulation, tolerance and volatilization.	2009	[35]
Review of knowledge of the role of Se in environmental sciences and discusses potential treatment options of Se contaminated waste streams.	2009	[54]
Review of plant Se metabolism and discussion of new insights into plant Se tolerance and hyperaccumulation mechanisms.	2010	[55]
Summarizes the knowledge on important organo-Se and Te species in environmental compartments, and identifies gaps and uncertainties in past and current analytical methodology.	2010	[14]
Discusses how Se phytoremediation may have ecological implications, particularly how high Se levels in plants affect herbivory.	2011	[56]
Review of how Se hyperaccumulators may profoundly affect their local ecosystem, by altering soil Se speciation and distribution and by selecting against Se-sensitive and for Se-resistant ecological partners.	2012	[57]
Overview of major aspects of volcanic–derived Se, with focus on processes in soils and aquifers.	2012	[41]
Discusses the role and importance of Se as selenoproteins in supplementing human diet.	2013	[24]
Se availability in semiarid soils and discusses how micronutrients can be effectively managed through the recycling of organic matter.	2013	[58]
Reviews studies on sorption of sorption, speciation and bioavailability of Se in non-flooded and wetland soils, incl. impact of redox/pH conditions, metal oxides, and organic matter.	2014	[59]
Summarizes what is known about plant metabolism of Se, and how plants may be used to clean up Se from agricultural drainage water.	2014	[60]
Summarizes the abundance and forms of Se in rocks, soils, sediments and natural waters and pathways and of Se in the natural environment	2014	[42]
Reviews the biogeochemistry of Se in the natural environment, in terms of variation of speciation with pH, redox conditions, sorption and interactions with natural organic matter.	2015	[61]
Overview of the role of Se-respiring bacteria (SeRB) in the biological Se cycle, their ecological role as well as Se biomineralization mechanisms and environmental biotechnological applications.	2015	[62]
Reviews what is known about plant metabolism of Se, molecular mechanisms of Se tolerance and (hyper) accumulation, achievements in plant genetic engineering of Se metabolism, and briefly touches on evolutionary and ecological aspects of plant Se accumulation.	2015	[63]
Discusses the function of Se in plant and human nutrition and the progress in the genetic engineering of Se metabolism to increase the levels and bioavailability of Se in food crops.	2015	[64]

### 3.1. Geogenic Sources of Soil Selenium

In areas where the underlying bedrock is high in Se, elevated Se concentrations can be found in soils as a consequence of weathering, leaching and transport of Se into soils. Examples of high Se regions are areas containing black shales, which are sedimentary rocks high in carbon and metal content [42,65,66,67,68,69,70]. Ireland contains some of the most Se-rich soils in the world derived from black shales, with levels up to 1250 mg·kg^−1^ [16]. Shales are also the primary source of high Se soils found in China [71,72,73], in arid regions in the west and southwest USA, e.g., in the central valley in California [74] and in the Colorado River Basin [75], as well as in South Dakota and Wyoming [76]. Although weathering and transport of Se from alluvial deposits are natural processes, release of toxic levels of geogenically-derived Se into the environment can additionally be triggered by human actions [77]. For example, Se contamination in the Kesterson reservoir (San Joaquin Valley, California), which seriously affected aquatic organisms and birds [78,79,80], has been the direct effect of the accumulation and evaporation of agricultural drainage water enriched in geogenic Se. Another source of Se to soils that is related to human activities are dusts in the vicinity of coal burning sites [81,82].

### 3.2. Atmospheric Sources of Soil Selenium

Although geogenic sources may be a major local source of Se to soils, a comparison between the average Se content in soils (ranging from 0.01 to 2 mg·kg^−1^, world mean 0.4 mg·kg^−1^, [13,83] and the average Se concentration in the Earth’s crust (0.05 mg·kg^−1^, [84] indicates that a surface source such as atmospheric deposition may play an important role in the global distribution of Se in soils. Sources and sinks of atmospheric Se have been reviewed in Wen and Carignan (2007) [40] (see Table 1). Global atmospheric Se budgets [85,86,87,88,89] indicated that atmospheric Se input fluxes are between 15.5 and 15.7 × 10^9^ g·year^−1^, of which ~60 and 40% are natural and anthropogenic emissions, respectively [40], although the contribution of natural emissions was found to be smaller (*i.e.*, 25%) in the northern hemisphere due to anthropogenic emissions [90]. Natural emissions include crustal weathering, sea spray, volcanic eruptions, and biological activity in the marine and continental biosphere **(**see Figure 1), while anthropogenic emissions include fossil fuel combustion, non-ferrous metal production and manufacturing ([40] and references therein). The main estimated anthropogenic source is coal combustion (3 × 10^9^ g·year^−1^ [86]; 1.83 × 10^9^ g·year^−1^ [87]) and the predominant natural atmospheric Se source may be the marine biosphere via biomethylation of Se by marine organisms [40,91] (see chapter 7).

#### Marine-Derived Atmospheric Selenium

Global biogenic emissions of sulfur (S), which has similar chemical properties as Se, significantly contribute to the global S cycle and may even affect global climate [92]. Even though the contribution of biogenic Se emissions to its global atmospheric budget is around 3 to 4 orders of magnitude smaller than for S emissions (approximately 6.5 × 10^9^ g Se year^−1^ [40] *versus* approximately 35 × 10^12^ g S year^−1^ [93], a comparison of these fluxes with the average concentrations in seawater (8.9 × 10^−1^ g L^−1^ S and 1.6 × 10^−7^ L^−1^ Se) [47] illustrates that Se is up to 3 orders of magnitude more enriched in the volatile phase than S. This relative enrichment of Se over S is corroborated by direct measurements of methylated Se and S species in seawater [91] and in a pristine wetland [94] by elemental analysis of aerosols [45,95]. Although the mechanisms underlying this enrichment of Se over S (e.g., selective biological uptake of Se over S) remain unelucidated and require further study, field measurements of biogenic Se species in the marine environment and global-scale extrapolations of these observations indicate that biogenic Se emissions from the marine environment may indeed be larger than previously estimated (28.5 × 10^9^ g Se year^−1^ [96] and 35 × 10^9^ g Se year^−1^ [91] compared to 0.4–9 × 10^9^ g year^−1^ [86,88]). However, most studies that measured biogenic Se concentrations were conducted during algal blooms and extrapolated global fluxes may therefore be overestimated.

### 3.3. Atmospheric Selenium Deposition

In contrast to global estimates of atmospheric Se emissions, global estimates of atmospheric Se deposition are not available. In the region between 30°N to 90°N, Ross (1985) [90] estimated fluxes of 36–100 × 10^8^ g Se year^−1^ and 5.5–26 × 10^8^ g Se year^−1^, for wet and dry deposition, respectively. Furthermore, Cutter (1993) [97] estimated a deposition of 2.2 × 10^9^ g Se year^−1^ over the North Atlantic Ocean, which comprises ~12%–18% of total global emissions as estimated by Mosher and Duce, 1987 [86]. However, most studies on atmospheric Se deposition are based on locally collected precipitation and aerosol samples and therefore do not account for spatial and temporal variation in atmospheric Se deposition.

Local studies carried out in the US (Chesapeake Bay, Massachusetts Bay), China (Mt Heng), Japan and the UK (various sites) have reported wet Se deposition fluxes between 140 and 502 μg·m^−2^·year^−1^ [98,99,100,101,102], whereas a dry deposition flux of 2 μg·m^−2^·year^−1^ and 140 μg·m^−2^·year^−1^ was estimated in the UK and Japan, respectively [99,102]. These deposition fluxes are roughly the same order of magnitude as emission fluxes from terrestrial systems (see Section 7.2). The measured fluxes of wet deposition do not, to a large extent, depend on the locations where they were measured, although coastal locations were generally characterized by higher fluxes. For example, coastal deposition of Se has been found in Norwegian forest soils, which shows a distinct decrease in Se concentration with increasing distance from the ocean [103]. Recently, Blazina *et al.* (2014) [104] used sediment sequences from the Loess Plateau in central China to show that over the last 6.8 million years rainfall likely controlled Se distributions in these sediments. This suggests that precipitation may be an important factor in controlling the broad-scale Se distribution in soils in monsoonal China. Further research is required to understand to what extent precipitation determines large-scale soil Se patterns in other parts of the world.

### 3.4. Anthropogenic versus Natural Sources of Atmospheric Selenium

Both anthropogenic and biogenic sources of atmospheric Se may contribute to Se levels in surface environments (see Figure 1). For example, anthropogenic sources of atmospheric Se were identified as an important source of Se to soils based on simultaneous decreases in Se levels in UK herbage and reductions in industrial emissions [105]. Similarly, water, soil, dust, air and locally produced food analyzed around the largest coking area in China were found to be enriched in Se [106], and aerosol samples collected over the North Pacific Ocean were concluded to be Se-enriched from Asian coal combustion activity [107]. On the other hand, Se concentrations measured in aerosols collected over the mid-North Pacific Ocean were concluded to originate from a dominantly biogenic input from the Bering Sea [86]. These examples indicate that the dominant sources of Se in the atmosphere (and thus the surface environment) may be highly variable depending on location and time. As a result, the extent to which different Se sources contribute to crop Se levels in different regions of the world is largely unknown.

#### Quantifying Se Sources and Sinks in Soils

Few studies systematically investigate the Se mass balance in soil/plant systems. As a result, the extent to which different Se sources contribute to plant Se content in different regions of the world is largely unknown. Nevertheless, the contribution of different Se input pathways into the soil was recently studied in a Se-rich area of China (Fujiang River Basin, Sichuan Province). The authors calculated annual average Se input and output fluxes of 20.45 g·ha^−1^·year^−1^ and 2.58 g·ha^−1^·year^−1^, respectively, and concluded that precipitation and infiltration were the main input and output pathways, respectively [108]. Further mass balance studies of Se in soils are required to i) quantify the extent to which different Se sources contribute to the Se content of agricultural systems, ii) understand how the different sources affect the Se speciation in soils, and iii) how much Se is retained in the soil and how much is removed via leaching, volatilization and crop harvest. Recently, the leaching and shallow subsurface-transport potential of agricultural contaminants (e.g., nitrate and steroids) has been evaluated utilizing lysimeters and tile drains beneath agricultural fields [109,110,111]. Such systems could be useful for quantifying the leaching potential for Se from soils. Only when we can better understand Se inputs and retention in soils we will be able to make predictions on future Se status in soils. There is still limited information on how atmospheric Se deposition affects the distribution of Se in the terrestrial environment. Due to the large mobility of atmospheric Se, understanding the role of atmospheric Se deposition in soil/plant systems is essential.

Stable Se isotope analysis is a promising tool to investigate redox reactions and to distinguish between potential Se sources. Johnson (2004) [50] noted that microbial reductions yield fractionations less than 5‰, whereas abiotic processes yield fractionations greater than 7.3‰, suggesting that it may be possible to distinguish microbial from abiotic reduction. Assimilation by higher plants, Se(IV) oxidation, sorption, and biological volatilization induce little or no isotopic fractionation [50]. In the last decade, analysis of Se isotopic fractionations to study geochemical processes and sources were applied to black shale-hosted Se-rich deposits in China [112,113,114], USGS shale standards [115], black shale samples from Australia and Canada [116] and sediments and soils [117,118]. A promising development is the ability to conduct stable Se isotope analysis of volatile organoselenides (after chemotrapping) [119], which may help distinguish biogenic atmospheric Se sources from anthropogenic sources.

## 4. The Mobility and Bioavailability of Soil Se

While broad and regional scale processes are important for governing the global distribution of Se, the local distribution of Se in terrestrial environments is governed by small and micro scale processes related to mobility and bioavailability (e.g., speciation, solubility, partitioning, uptake, *etc.*) [120]. Mobility and bioavailability are interrelated but not necessarily synonymous. For example, highly immobile chemicals are unlikely to be bioavailable but highly mobile constituents may or may not be bioavailable depending on specific plant uptake mechanisms. Similarly, following uptake of bioavailable constituents, plants can facilitate the regional redistribution of Se via volatilization to the atmosphere but can also decrease mobility by accumulating Se into plant tissues.

### 4.1. Factors Affecting Se Mobility in Soils

Over the last several decades, the chemical processes affecting Se mobility in the environment have been well characterized [13]. In the environment, Se can exist in the (-II), (-I), (0), (IV), (VI) oxidation states, and in general, Se solubility, and therefore mobility, increases with increasing redox potential (*i.e.*, more oxidizing conditions). Soil Se is mainly inorganic but it can also be present in organic forms, e.g., as complexes with organic matter and incorporated into organic or organo-mineral colloids [13,59,121,122] (Figure 1). Based on their acid dissociation constants (pKa), seleneous acid (H_2_SeO_3_) and selenic acid (H_2_SeO_4_) are anionic under common environmental conditions [48], e.g., as selenite (SeO_3_^2−^) and selenate (SeO_4_^2−^). Selenium in the (-II) oxidation state exists as gaseous hydrogen selenide, as well as numerous metallic selenides (e.g., iron selenide), which are the most insoluble forms of Se [123,124]. A number of studies have reported that soil bacteria can form (nano)-sized Se(0) from selenate and selenite [125,126,127,128,129] but it is unclear if microbial production of (nano) Se(0) is important in natural environments and what the fate of biogenic nanoparticular Se is in soil systems. In addition, volatile organic forms of Se such as dimethyl selenide (DMSe) and dimethyl diselenide (DMDSe) may be present in soils [49].

Due to the anionic form of Se, electrostatic forces are likely to dominate sorption-interactions in soil [48]. Selenium can be sorbed to layered double hydroxides or anionic clays through electrostatic interactions, which are highly pH sensitive [130,131,132,133]. As pH decreases, Se adsorption increases due to fewer negatively charged clay surfaces and sesquioxide edges, resulting in stronger electrostatic interactions [134]. Therefore, the mobility and bioavailability of inorganic Se in the environment increases with increasing pH, as well as with decreasing clay and iron oxide content in the soil [130,135,136].

#### The Role of Organic Matter in Se Retention by Soils

Soil organic matter (OM) is known to influence the retention of Se in soils [137]; however, the mechanisms of Se-OM interactions are poorly understood. Three hypotheses explaining the OM mediated retention of Se are generally discussed: (i) OM has increased sorption sites, which facilitates direct complexation with Se [137,138,139,140,141,142]; (ii) indirect complexation via OM-metal complexes [137,143,144,145,146]; or (iii) microbial reduction and incorporation into amino acids, proteins, and natural organic matter [141,144,145,147,148]. Depending on the type of binding, Se may be easily mobilized (e.g., through pH adjustment) or immobilized (e.g., covalent incorporation to OM). In general, however, the molecular structure and speciation of Se bound to OM is still unclear [149]. Many studies have reported large amounts of unidentified Se fractions in soil extracts, up to 100% [150,151,152,153]. Organo-Se compounds could make up a major fraction of the unidentified species. Therefore, further studies will be required to identify unknown Se species and ultimately learn more about the fate of Se in the environment [154].

**Figure 2:**
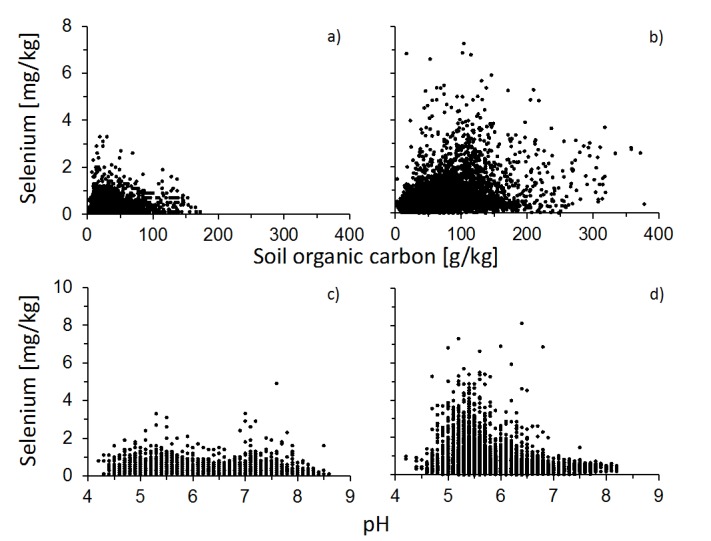
Scatter plot illustrating large-scale nonlinearities between soil Se and soil organic carbon (**a**,**b**) and soil pH (**c**,**d**) in the United States (a,c) and Western Europe (b,d). Soil Se analyses were published by the USGS (2014) [155] and Reimann *et al.* (2014) [156] and the organic carbon and pH data were published by Hengl *et al.* (2014) [167].

While general trends have been reported to describe the relationship between organic matter and pH in soils, few studies have examined Se accumulation trends in soils across broad (*i.e.*, nation or continental) scales. Figure 2 illustrates the relationship between OM, pH, and soil Se across the United States [155] and western Europe [156] and clearly shows that the relationships between Se and chemical soil properties over large spatial scales are complex. This complexity is likely a result of increased environmental heterogeneity compared to small-scale experiments, for example due to different Se source contributions, resulting in highly nonlinear patterns. The factors driving the relationships between Se, soil, OM and pH over large spatial scales is not clear and further studies are required to elucidate the role of soil and climate variables in driving these relationships.

### 4.2. Soil Factors Affecting Uptake of Se by Plants

**Figure 3:**
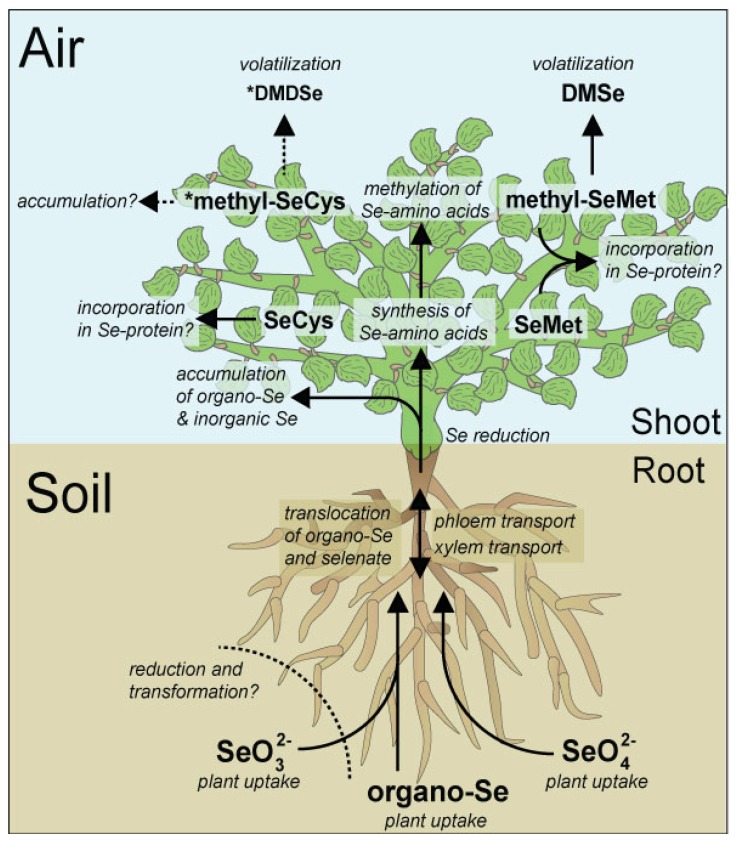
Overview of major pathways of Se at the soil-plant-atmosphere interface. The transport- and transformation processes of Se in higher plants are indicated in italics at the corresponding arrows. Compounds indicated with an asterisk are thought to only occur in Se-accumulator plants.

Depending on the soil type, selenate and selenite are bioavailable to plants [123,124,157,158] and factors affecting Se mobility in soils thus also affect plant bioavailability (see Figure 3 for a schematic diagram of Se cycling at the plant-soil interface). Nevertheless, Se bioavailability to plants is poorly characterized due to the lack of studies that quantify the collective factors known to affect Se mobility and uptake in the environment. Most studies have been conducted in controlled environments (e.g., pot and greenhouse experiments), which oversimplifies the chemical, physical and biological processes present in nature. Increases in pH are commonly mentioned as the most important driver leading to higher Se concentrations in crops [158,159]. As with mobility, uptake is generally positively correlated with pH but this relationship is most apparent near pH values where selenate adsorption on iron oxides ceases (~pH 6.5) [159]. The relationship between pH and Se uptake varies depending on other factors including soil type: increases in pH have been correlated to higher Se uptake in sandy and loamy soils but not for clay soils [159,160,161]. Furthermore, in soils rich in OM, Se uptake was shown to decrease with increasing pH [134,161]. Such studies illustrate that trends in Se uptake largely depend on the type of soil.

#### 4.2.1. The Role of Soil OM in Plant Se Uptake

In addition to pH, Se uptake is greatly affected by OM. Because OM increases Se retention in soils, it would be logical to assume that bioavailability should be negatively correlated with OM. As expected, several studies have observed decreased Se uptake with increasing OM [135,161,162]; however, strong positive correlations between OM and Se uptake have also been reported [119]. Conversely, Johnsson (1991) [161] reported an increase in Se uptake under moderate OM concentrations (5% to 11%) but a decrease in uptake when OM was >11%. Various hypotheses have been proposed to explain the seemingly contradictory findings between Se uptake and soil OM, which is likely to improve soil structure and promote oxidizing conditions thus increasing bioavailability [161,163]. Conversely, differential partitioning of Se to fulvic (FA) and humic (HA) acids may alter Se bioavailability in soils. Qin *et al.* (2011) [149] reported that Se was weakly partitioned to FA but was relatively strongly bound to HA. Thus, soils with high FA content could increase bioavailability by acting as a source of Se, which is supported by observations [164]. Finally, increases in OM have been proposed to increase microbial activity and therefore promote the incorporation of inorganic Se into biomass [144,165,166].

#### 4.2.2. The Role of Combined Soil Properties in Plant Se Uptake

While the effect of individual factors on Se uptake is generally known in controlled experiments, the combination of factors affecting Se uptake in natural systems is largely unknown. For example, Liu *et al.* (2015) [168] reported that S addition to soils decreased pH and increased Se partitioning to Fe/Mn oxides, as well as to OM, all of which decrease Se uptake, but the dominant mechanisms governing the Se uptake in wheat were not identified. More sophisticated analyses that are capable of differentiating the importance of individual factors, as well as interactions among factors, are required for identifying the dominant processes that drive Se mobility and bioavailability within the environment. In addition to greenhouse or plot scale experiments, comprehensive medium/large scale observational studies are needed in combination with multivariate statistical analyses. Such analyses facilitate the examination of interactive effects of various combinations of factors. Furthermore, such studies should be conducted in a variety of ecosystems with different plant communities and different soil physicochemical properties to identify how the dominance of processes that govern Se bioavailability shifts along environmental gradients. Such knowledge is critical for manipulating the Se status of edible crops, as well as for optimizing phytoremediation efforts.

When all factors are held constant, the Se status of crops is linearly related to the soil Se concentrations [169,170], but this linear relationship may be a simplification of the complexities observed in nature. Overall, mobility and bioavailability are not only interdependent but are also dependent on interactions between soil pH, clay content, and ionic composition (e.g., iron, sulfate, phosphate, *etc.*) in addition to the microbial community and climatic factors (e.g., humidity and evapotranspiration; [166,171,172]). Collectively, interactions among all these variables fundamentally result in a highly non-linear relationship between Se and mobility/bioavailability, which makes identifying the dominant processes governing this interaction difficult. Relatively recently, several standardized, large-scale (e.g., nation-wide) soil geochemical analyses have been conducted within China, Europe, the US, the UK, and others [155,156,173,174]. Such datasets could be coupled with other broad scale data (e.g., climate and soil physicochemical properties) to understand how Se bioavailability and mobility change along such environmental gradients. These datasets can further be used to explore relationships between Se and other soil parameters over large spatial scale.

## 5. Mechanisms of Se Uptake and Metabolism

### 5.1. Selenium Uptake by Plants

#### 5.1.1. Uptake of inorganic Se

Although Se has not been shown to be an essential micronutrient in higher plants, plants differentially take up various Se species. These differences in uptake drastically alter the bioavailability of Se to plants, and may have a profound influence on the redistribution of Se in the environment. Plants may take up both inorganic and organic Se via active membrane transport (Figure 3) while the role of simple diffusion is limited [34,175]. The active transport of Se appears to occur via shared transporter-proteins: selenate may be actively internalized by sulfate transporters [34,117,176] and selenite is likely internalized via phosphate transporters [34,158,159,160,161,162] and possibly Si-transporters (e.g in rice [177]. Uptake of Se in the presence of sulfate and phosphate is suppressed [34,167,178,179]. Conversely, selenate and selenite uptake is enhanced by low S and P concentrations in soils, respectively [175] causing plants to increase the expression of sulfate and phosphate transporter genes in order to compensate for the lower availability of these nutrients. As a consequence, both selenate and selenite are taken up more readily when present in solution in the absence of sulfate and phosphate. Response to lower availability of nutrients has been reported to be more pronounced for selenate than for selenite uptake, which could be due to a different level of gene up-regulation in reaction to P or S starvation and/or to the lower affinity of the phosphate transporters for selenite compared with that of sulfate transporters for selenate [175].

#### 5.1.2. Uptake of Organic Se

In addition to inorganic Se, plant uptake of organic Se is known to occur and has been reported at much higher rates (20–100 fold greater) than the uptake of inorganic species [148,180,181]. Evidence suggests that amino acid transporters are important [181]. To date, no Se-specific uptake mechanisms have been reported but these may exist in Se hyperaccumulator species since selenate uptake is much less inhibited by sulfate, and they typically have a higher Se:S ratio compared to their growth substrate and to other plant species (see chapter 6). Given the need to optimize the Se status in crops in low-Se areas, the importance of understanding plant physiology and the cell-membrane transport of Se cannot be overstated.

### 5.2. Uptake of Inorganic and Organic Se by Other Organisms

Similar to plants, many microalgae and bacteria take up both inorganic and organic Se via active membrane transport using shared transporter-proteins. There are indications that selenate is internalized via sulfate transporters [182] and selenite may be internalized with phosphate-transporters (e.g., in algae [183,184] and yeast [185]. Selenite may also be internalized via monocarboxylate transporters (e.g., in yeast [186]). Specific Se influx transporter proteins for inorganic Se have not been identified but have been suggested to exist [187]. The membrane-transport of organo-Se species SeMet and SeCys has been investigated in animal and human cells and also occurs via shared transporters [188], *i.e.*, methionine [189] and cysteine [190] transporters. In general, higher animals and humans directly acquire organic Se compounds (SeMet and SeCys are the major Se species in the diet) but they can also internalize inorganic Se [9]. In contrast, plants and microorganisms may predominantly take up inorganic Se [34,191] and therefore need to synthesize their own Se-amino acids, which probably occurs through the same metabolic reactions involved in the synthesis of S amino acids [192] discussed below.

**Figure 4:**
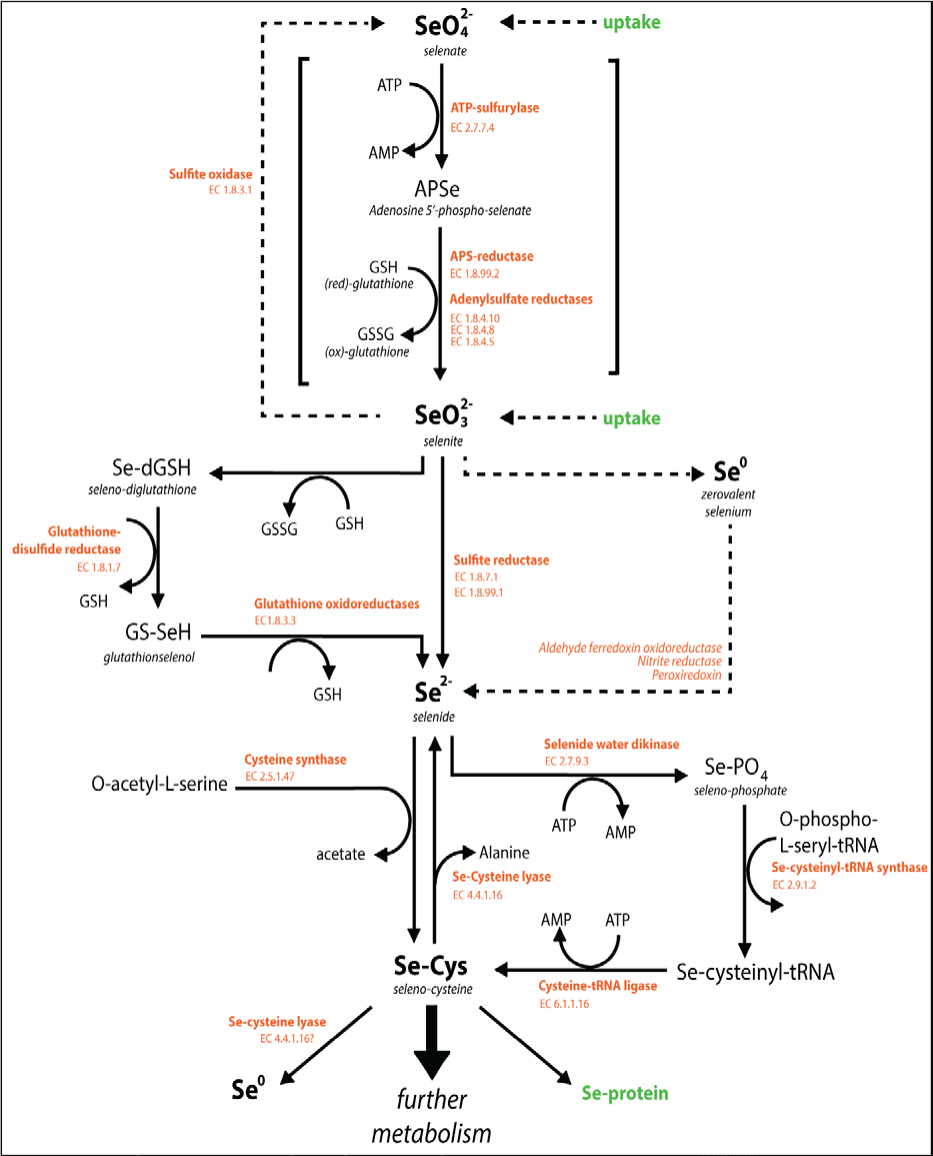
Schematic diagram of the biochemical reactions in the uptake and metabolism of Se in plants and microorganisms. Identified catalyzing enzymes and their Enzyme Commission (EC) numbers are indicated in orange at the corresponding reactions. Major (intermediate) compounds are indicated in bold. Information was compiled from previous reviews [11,34,49,55,117,185,193,194,195].

#### Selenium Metabolism

The uptake of Se and synthesis of Se amino acids has been investigated in algae [193,196] and plants [34,117]; a schematic overview is given in Figure 4. Briefly, intracellular selenate is reduced to selenite via activation with ATP-sulfurylase, and selenite may be further reduced to selenide, which may occur both enzymatically and non-enzymatically [34]. Subsequently, selenide is incorporated into SeCys via the coupling with O-acetylserine that is catalyzed by the enzyme cysteine synthase (Figure 4). In turn, SeCys may be further metabolized into SeMet via the methionine cycle, which includes enzymatic transformation of SeCys to Se-cysthationine, Se-homocysteine and finally SeMet [34]. The amino acids SeMet and SeCys are pivotal Se molecules because they have direct antioxidant functions and fulfill a number of essential physiological functions through deliberate incorporation of SeCys into Se-proteins [197,198,199]. On the other hand, non-specific incorporation of Se-amino acids (SeCys) proteins is a major cause of Se toxicity [200]. Furthermore, the Se-amino acids may be considered precursors of methylated, volatile Se species that can be emitted to the atmosphere. Biomethylation will be further discussed in chapter 7.

## 6. Selenium Hyperaccumulation by Plants

Some plant species native to seleniferous soils accumulate Se to levels typically 100-fold higher than other vegetation: upwards of 1000 mg·kg^−1^ (dry weight) and up to 15,000 mg·kg^−1^ (dry weight); these are considered Se hyperaccumulators [201]. Selenium hyperaccumulation has been reported in ~30 species in the families Brassicaceae (*Stanleya*), Fabaceae (*Astragalus*) and Asteraceae (*Xylorhiza*, *Oonopsis*, *Symphyotrichum*) [202,203]. Hyperaccumulation is found in the more evolutionary derived taxa within a family [204,205] suggesting that Se hyperaccumulation is a derived trait that evolved independently in different families, perhaps under similar selection pressures. It is intriguing that Se hyperaccumulators accumulate an element that is not essential for higher plants [206], and that they not only tolerate but even grow better at tissue Se levels that are lethal for other plant species.

### 6.1. Physological and Ecological Benefits

Plant accumulation of Se may have a physiological benefit since Se can have a growth-promoting effect in many species and therefore is considered a beneficial element [207]. Selenium hyperaccumulators show a particularly strong positive growth effect when supplied with Se, which may exceed a 2-fold increase in biomass production [57]. The mechanism by which Se exerts this positive physiological effect is largely unclear; however, Se has been shown to upregulate antioxidant responses in plants, which may allow them to withstand a variety of oxidative stresses [27]. Selenium accumulation also confers several ecological benefits to plants. There is ample evidence to indicate that Se protects plants from herbivory [208,209,210,211,212,213,214,215,216] and some evidence that it may also protect from pathogens and offer a benefit via elemental allelopathy (for a review see El Mehdawi and Pilon-Smits, 2012 [57]). The protective effects of Se have been found not only for hyperaccumulators but also for other plants at lower Se concentrations [208,216]. Thus, Se hyperaccumulation may have the additional benefit of decreasing competition from other vegetation. Therefore, it appears that Se offers the plant multiple benefits: protection from herbivores and pathogens, reduced competition from other plant species and also a physiological benefit through enhanced growth. These benefits likely have driven the independent evolution of Se hyperaccumulation in different lineages. There is no clear evidence that Se hyperaccumulation carries an evolutionary cost in terms of negative effects on mutualistic ecological partners [217,218].

### 6.2. Mechanisms of Plant Se Hyperaccumulation

Like other plants, hyperaccumulators use the sulfate uptake and assimilation pathway to metabolize Se, since Se is sufficiently chemically similar to S that most transporters and enzymes cannot distinguish S and Se analogues [219]. However, hyperaccumulators have been found to differ from non-hyperaccumulators in several ways. They have higher S levels than non-hyperaccumulators [217], indicative of an upregulated S/Se uptake system. Furthermore, selenate uptake by hyperaccumulators is not inhibited by high sulfate concentration [220,221,222], and hyperaccumulators also tend to have a higher Se/S ratio in their tissues compared to their growth medium [223]. This observation points to the presence of a transporter with enhanced selenate specificity relative to sulfate. Indeed, a high-affinity root sulfate transporter was found to be expressed at much higher levels in *S. pinnata* relative to *B. juncea* [179]. Hyperaccumulators also show evidence of enhanced S/Se assimilation to organic forms: the main form of Se accumulated by hyperaccumulator species is organic methyl-SeCys [224], while in related non-hyperaccumulators a large fraction of the Se remains in the inorganic form of selenite [225,226]. The mechanism underlying the enhanced S assimilation may be upregulated expression of genes involved in S/Se assimilation, particularly ATP sulfurylase and APS reductase [33,180]. Furthermore, hyperaccumulators have particularly high levels of SeCys methyltransferase, which converts SeCys to methyl-SeCys [227].

At the whole-plant level, hyperaccumulators differ from other plants in that they translocate more Se from roots to shoots via xylem transport and from leaves to reproductive organs via phloem transport [204]. They store Se mainly in young leaves and reproductive organs (pollen, ovules, seeds), within epidermal vacuoles [218,224]. The transporters involved in these sequestration processes remain to be identified. Non-hyperaccumulators mainly store Se in vascular tissues in leaves, and have higher Se levels in leaves than flowers [200,205]. The finding that hyperaccumulators store Se in their epidermal tissues as methyl-SeCys may explain their Se tolerance: this form of Se does not cause oxidative stress (like selenate and selenite), does not get incorporated into proteins (like SeCys and SeMet), and can safely be accumulated. While not toxic to the plant, methyl-SeCys may become toxic to herbivores and pathogens upon ingestion and demethylation and can therefore serve a defensive function [228]. The preferential sequestration of methyl-SeCys in reproductive organs and peripheral tissues may optimize its defensive effect. In hyperaccumulators, MethylSeCys can also be converted to DMDSe (see Figure 3 and chapter 7), an odorous volatile Se compound, which may contribute to herbivore deterrence [34].

### 6.3. Ecological Impacts of Hyperaccumulators on Seleniferous Areas

Furthermore, it is likely that Se hyperaccumulators affect Se distribution and speciation in soil: they are perennials with deep root systems that scavenge a large volume of soil for Se and translocate it to the aboveground organs. In the process, inorganic Se is converted to organic methyl-SeCys. In the fall, part of the sequestered Se is deposited on the soil surface as litter, and some is remobilized to the root [229]. Over time, deposition of Se-rich litter may enrich the soil around hyperaccumulators with Se, creating Se hot spots [216]. The organic Se-enriched soil and litter around hyperaccumulators, as well as the Se-rich organs of the living plant itself may be utilized by a variety of Se-tolerant organisms, thereby facilitating Se transport into the food chain with subsequent cycling in the local ecosystem [57,216].

## 7. Biomethylation of Se

**Figure 5:**
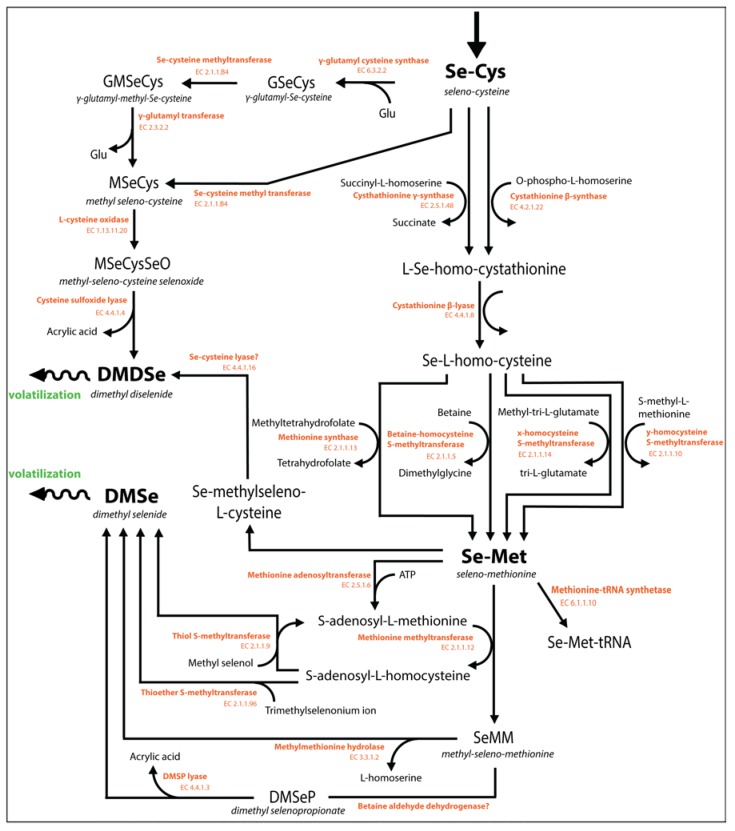
Schematic of the biochemical reactions involved in Se methylation in plants and microorganisms. Identified catalyzing enzymes and their EC numbers are given in orange at the corresponding pathways. Major (intermediate) compounds and volatile end-products are indicated in bold. Information is compiled from previous reviews [11,34,49,55,117,184,193,194,195].

The term ‘biomethylation’ includes all chemical processes in biological systems that involve the transfer of a methyl group [196]. At the cellular level, methyl-transferase enzymes catalyze the transfer of a methyl group from an organic substrate to an acceptor molecule, which can yield both volatile and non-volatile methylated products [230]. When volatile methylated species are formed, these may diffuse out of the cell and be released into the atmosphere. Therefore, biomethylation is a mechanism that likely enhances the mobility of Se in the environment and can lead to environmental redistribution of Se. The most commonly detected volatile organo-Se compounds in environmental samples and as microbial and plant products are dimethyl selenide (CH_3_SeCH_3_, DMSe), dimethyl selenenyl sulfide (CH_3_SeSCH_3_, DMSeS), and dimethyl diselenide (CH_3_SeSeCH_3_, DMDSe) [49,231]. The majority of information on the pathways of Se methylation is based on research conducted with (Se-tolerant) plants [36,37] and has been extensively reviewed [49,55]. A schematic overview of the biochemical reactions involved in Se methylation is given in Figure 5. Biomethylation of Se has been observed in all phylogenetic kingdoms [195], but not all organisms appear to methylate Se [49]. Organisms that have the physiological tools to perform Se biomethylation include bacteria [197,232], fungi [233], photosynthesizing microalgae [234] and plants [34], animals [235,236] and humans [237,238].

### 7.1. The Mechanism of Se Biomethylation

The microbial methylation of arsenic occurs through a single, generally accepted mechanism: the ‘Challenger mechanism’. This mechanism involves an alternation of reduction and methylation reactions, involving S-adenosyl methionine (SAM) as the methyl-donor compound [194,230,239]. Although originally thought to be valid for the biomethylation of Se as well, the Challenger methylation mechanism has been repeatedly updated and adjusted, based on discoveries of new Se metabolic pathways and compounds [240,241], as reviewed by Chasteen and Bentley 2003 [49]. Currently, the Se amino acids SeMet and SeCys are considered central metabolites in the pathway of Se methylation (see Figure 5).

#### 7.1.1. Selenium Methylation by Plants

Pathways of Se metabolism and methylation are known to vary between Se hyperaccumulator and non-hyperaccumulator plants [34]. In plants, the amino acids SeMet and SeCys can be methylated to form methyl-SeMet and methyl-SeCys, respectively [55]. This occurs through the activity of methyl-transferase enzymes that catalyze the transfer of a carbocation (CH_3_^+^, donated from S-adenosyl methionine or methyltetrahydrofolate) [34,230] to the Se atom on the respective amino-acid. Alternatively, SeCys may be converted into methyl-SeCys via glutamination into γ-glutamyl-SeCys, subsequent methylation into γ-glutamyl-methyl-SeCys and finally deglutamination (Figure 5). The methylated amino-acids methyl-SeCys and methyl-SeMet can be regarded as the direct precursors for the volatile Se species DMSe and DMDSe, respectively. The first methylated Se-amino acid, methyl-SeCys, may be oxidized to methyl-SeCys-selenoxide by the action of cysteine-oxidase and subsequently be cleaved by cysteine sulfoxide lyase to yield the volatile methyl selenol (MeSeH) and DMDSe (Figure 5). The second methylated Se-amino acid, methyl-SeMet, may be directly cleaved to DMSe by the action of the enzyme methyl-methionine hydrolase. Alternatively, methyl-SeMet may be transformed into dimethyl-selenopropionate (DMSeP), which is a direct analogue of dimethyl sulfoniopropionate (DMSP) [180,242], and ultimately be cleaved by DMSP-lyase to yield DMSe and acrylic acid. The compound DMSP is a known precursor of volatile dimethyl sulfide (DMS, the direct analogue compound of DMSe) and fulfills osmoprotective- and other physiological roles in marine algae and bacteria [243]. Although DMSP is abundant in marine algae and bacteria, its Se analogue has only been identified in Se-accumulator plants in very small amounts [180]. Furthermore, the reactions and enzymes involved in the synthesis of DMSP (and likely DMSeP as well) are still unknown for many organisms [244].

#### 7.1.2. Formation of DMSe *versus* DMDSe

The production of DMSe has been found in many environments, usually at higher rates than DMDSe (see Section 7.2), with exception of the production of DMDSe by Se-tolerant hyperaccumulator plants [245]. Traditionally, biomethylation is often regarded as a detoxification mechanism since the methylated volatile Se species are less toxic than their inorganic precursors [246] and because volatilization offers a loss-mechanism of excess intracellular Se. The abundance of DMSe suggests that the pathway of DMSe synthesis via methyl-SeMet is the most common way for Se methylation by plants and soil microbes. In turn, the formation of DMDSe via methyl-SeCys in Se-tolerant plants has been explained by the fact that these plants have higher intracellular Se concentrations and thus are required to detoxify more Se (DMDSe may be regarded as a more efficient detoxification product than DMSe). This hypothesis is supported by the fact that methyl-SeCys, the precursor for DMDse, is not incorporated into proteins and may therefore be a means for Se-accumulator plants to safely store Se [247] (Figure 3). However, the amino-acids SeMet and SeCys may be continuously interconverted via the methionine cycle and transsulfuration [55] so that either of the methylation pathways of these amino acids (and subsequent formation of the volatile species DMSe and DMDSe) may become alternately prevailing. In addition, transformation of volatile Se compounds may occur after release from the cell (*i.e.*, by demethylation-, (photo)-oxidation- and chalcogenide exchange reactions) so that the observed volatile speciation in the environment may not be a direct reflection of the cellular mechanism operating [248]. Therefore, further research is required to resolve the exact biochemical mechanisms by which different organisms synthesize different volatile Se species. Such information on the production mechanisms is particularly important considering that the different volatile Se species may have different atmospheric lifetimes [40].

### 7.2. The Occurrence of Methylated Se in Soils, Water and Air

The biogenic volatile Se compounds DMSe and DMDSe have been found in soils [240], freshwaters [249,250], marine waters [97,251] and air [97,252]. However, quantitative measurements of volatile, methylated Se compounds in the natural environment are relatively scarce. Relatively high concentrations of DMSe and DMDSe have been measured in surface waters in Se-rich systems: up to 2.1 nM DMDSe has been detected in contaminated lake water [253] and concentrations of 130–943 nM and 13.2–38.3 nM DMSe and DMDSe, respectively, have been reported in Se-rich San Joaquin groundwater [250]. Concentrations of up to 9 nM DMSe and 11 nM DMDSe have been measured in river sediments [254], while in river water up to 37 pM DMSe has been quantified [255]. Quantification of volatile Se has also been conducted in pristine estuarine waters: Tessier *et al.* (2002) [256] report concentrations of 0.2–99.5 pM DMSe and up to 1.8 pM DMDSe and Pécheyran *et al.* (1998) [251] concentrations of 0.65–6.49 pM DMSe and up to 2.39 pM DMDSe in estuarine surface waters. In the open marine environment, concentrations of DMSe and DMDSe are usually lower: 0.14–4.73 pM DMSe and 0.02–0.11 pM DMDSe in the North Atlantic [91] and 0.06–0.64 pM DMSe and 0.07–0.26 pM DMDSe in the Mediterranean [96]. The lowest concentrations of methylated Se compounds have been measured in air: 6–80 pmol·m^−3^ DMSe was measured in urban air [257], up to 22 pmol·m^−3^ DMSe and up to 2 pmol·m^−3^ DMDSe in air collected near rivers and streams in Belgium [252] and 0.3–1.2 pmol DMSe m^−3^ and 0.3–0.6 pmol·m^−3^ DMDSe in remote marine air [96].

In addition to DMSe and DMDSe, the volatile Se species MeSeH and the mixed Se-S species dimethyl selenylsulfide have been identified in natural waters [91,96,256,258]. Other volatile Se species such as dimethyl triselenide, ethylated-, mixed methyl-ethylated selenides and mixed Se-S species such as dimethylselenyl sulfide, dimethyl selenyldisulfide, dimethyl diselenyl sulfide have been identified in the headspace of Se-amended bacterial cultures [259,260] but never in natural environmental samples. It remains unclear by which mechanisms such volatile Se species can be produced.

#### 7.2.1. Measurements of Biogenic Se Emissions

Atmospheric emissions of Se may occur either directly from vegetation (e.g., through the plant leaf surface) or through the water-air or soil-air interfaces (Figure 1). Similar to measurements of Se emissions from the ocean (see Section 3.2), there is a limited number of studies that have quantified Se fluxes from terrestrial environments, especially in natural, pristine environments [94]. Methylation and volatilization of Se from soils has been of particular interest for bioremediation strategies in high-Se environments that rely on the activity of microbes and vegetation to volatilize Se and thereby reduce Se contamination in the surface environment [36]. Field emissions of methylated Se have been measured using flux chambers combined with trapping systems (e.g., acid traps or cold traps) [261]. Sensitive measurements of Se volatilization in laboratory settings have also been obtained by spiking soil and sediment samples with radioactive ^75^Se [262].

Although quantitative measurements are relatively scarce, the biogenic production of volatile Se compounds and the corresponding Se emissions have been assessed in a variety of environmental compartments including soils and sediments [37,262,263,264,265,266,267], plant leaves [268], peat bogs [95,261], estuarine waters [268], lakes [269] and in the marine environment [92,96]. A summary of observed biogenic production of volatile Se is provided in Table 2.

**Table 2:** Measurements of biogenic Se fluxes in lab and field experiments from different environmental systems (including seleniferous soils).

Reference	Production of Volatile Se	Experiment	Amendment	Environment	Se Content
(Lin, Cervinka *et al.* 2002) [266]	11–155 μg Se·m^−2^·d^−1^	Field	No	Soil	4.0 mg·kg^−1^
(Bañuelos and Lin 2007) [37]	25 μg Se·m^−2^·d^−1^	Field	No	Soil	4.78 mg·kg^−1^
(Bañuelos, Lin *et al.* 2005) [267]	3–56 μg Se·m^−2^·d^−1^	Field	No	Soil	3–8.2 mg·kg^−1^
(Wu and Huang 1991) [270]	180 μg Se·m^−2^·d^−1^	Field	No	Soil	1 mg·L^−1^
(Dungan, Stork *et al.* 2000) [271]	17–72 mg Se·m^−2^·h^−1^	Field	No	Soil	0.14 mg·kg^−1^
(Zawislanski 1996) [272]	20–200 μg Se·m^−2^·d^−1^	Field	No	Soil	50–180 mg·kg^−1^
(Lin, Hansen *et al.* 1999) [273]	117–125 μg Se·m^−2^·d^−1^	Field	No	Soil	4–8 mg·kg^−1^
(Bañuelos and Lin 2007) [37]	114–434 μg Se·m^−2^·d^−1^	Field	Yes	Soil	4.78 mg·kg^−1^
(Frankenberger W.T. Jr. 1995) [265]	72–1300 μg Se·m^−2^·d^−1^	Field	Yes	Soil	11.4 mg·kg^−1^
(Bañuelos, Terry *et al.* 2005) [36]	4–13.4 μg Se·m^−2^·d^−1^	Laboratory	No	Soil	0.15–13 mg·kg^−1^
(Wu and Huang 1991) [270]	65 μg Se·g_soil_^−1^·d^−1^ *	Laboratory	No	Soil	1 mg·L^−1^
(Martens and Suarez 2003) [274]	<1 μg Se·kg_soil_^−1^·d^−1^	Laboratory	No	Soil	10 mg·kg^−1^
(Moreno-Jiménez, Clemente *et al.* 2013) [275]	4–214 μg Se·kg_soil_^−1^·d^−1^	Laboratory	Yes	Soil	68 mg·kg^−1^
(Zieve and Peterson 1981) [276]	2 μg Se·kg_soil_^−1^·d^−1^	Laboratory	Yes	Soil	5 mg·kg^−1^
(Stork, Jury *et al.* 1999) [262]	<700 μg Se·kg_soil_^−1^·d^−1^	Laboratory	Yes	Soil	18.3–0.14 mg·kg^−1^
(Frankenberger and Karlson 1989) [277]	<1.1 mg Se·g_soil_^−1^·d^−1^	Laboratory	Yes	Soil	60 g·kg^−1^
(Dhillon, Dhillon *et al.* 2010) [278]	<32 ng Se·kg_soil_^−1^·d^−1^	Laboratory	Yes	Soil	1.7–31 mg·kg^−1^
(Karlson and Frankenberger 1988) [263]	<677 μg Se·kg_soil_^−1^·d^−1^	Laboratory	Yes	Soil	100 mg·kg^−1^
(Vriens, Lenz *et al.* 2014) [94]	0.11–0.12 μg Se·m^−2^·d^−1^	Field	No	Wetland	2.0 ± 0.4 mg·kg^−1^
(Vriens, Ammann *et al.* 2014) [261]	190–210 ng Se·m^−2^·d^−1^	Field	No	Wetland	2.0 ± 0.4 mg·kg^−1^
(Hansen, Duda *et al.* 1998) [279]	25–190 μg Se·m^−2^·d^−1^	Field	No	Wetland	4–31 μg·L^−1^
(Gao, Tanji *et al.* 2003)[280]	44–285 μg Se·m^−2^·d^−1^	Field	No	Wetland	10 μg·L^−1^
(Zhang and Moore 1997) [281]	0.1–60 μg Se·m^−2^·d^−1^	Laboratory	Yes	Wetland	2–11 μg·L^−1^
(Thompson-Eagle and Frankenberger 1991) [282,283]	0.1–10 μg Se·L_water_^−1^·d^−1^	Laboratory	Yes	Wetland	0.02–102 mg·L^−1^
(Thompson-Eagle and Frankenberger 1990) [282,283]	0.55–6.9 μg Se·L_water_^−1^·d^−1^	Laboratory	Yes	Wetland	14–2000 μg·L^−1^
(Thompson-Eagle and Frankenberger 1990) [282,283]	<18 μg Se·L_water_^−1^·d^−1^	Laboratory	Yes	Wetland	0.7–2.9 mg·L^−1^
(Calderone, Frankenberger *et al.* 1990) [284]	<25 μg Se·kg_sediment_^−1^·d^−1^	Laboratory	Yes	Sediment	<40.7 mg·kg^−1^
(Diaz, Johnson *et al.* 2008) [269]	<1.8 μg Se·m^−2^·d^−1^	Field	No	Lake	6.3 nM
(Amouroux and Donard 1997) [258]	0.002–0.5 μg Se·m^−2^·d^−1^	Field	No	Estuary	0.06–1.12 nM
(Terry, Carlson *et al.* 1992) [268]	15–350 μg Se·m^−2^·d^−1^ (m^2^ leaf area) * 0.2–2.5 mg Se·kg_plant_^−1^·d^−1^	Laboratory	Yes	Plant leaf	< 20 μM
(Amouroux and Donard 1996) [96]	0.002–0.23 μg Se·m^−2^·d^−1^	Field	No	Marine	~1 nM
(Fan, Higashi *et al.* 1998) [285]	<0.75 μg Se·L_water_^−1^·d^−1^	Laboratory	Yes	Marine	Up to 1 mg·L^−1^

* dry weight.

Recently, direct flux-chamber measurements of Se emissions in a pristine wetland showed that Se fluxes can be substantial: an average volatile flux of 0.12 mg·m^−2^·day^−1^ was measured [94]. Extrapolation of this flux to the global wetland area would equal a flux of 0.19–0.37 × 10^9^ g Se year^−1^ from wetlands, which is 5%–10% of the currently estimated marine Se volatilization. However, further field studies in naturally-low Se areas are critically needed to assess the importance of terrestrial emissions of Se.

#### 7.2.2. Limitations of Emission Data

Due to differences in experimental conditions (laboratory or field, amendment with Se and/or carbon *versus* natural Se levels) and differences in units (some Se emissions are reported per kilogram soil or sediment, while other fluxes are expressed per square meter of soil/air interface), a comparison between measured Se emissions is complicated. Nevertheless, the range of fluxes given in Table 2 illustrates that there is a high spatial and temporal variability in Se emissions (up to 4 orders of magnitude within comparable systems) caused by the fact that emissions of biogenic methylated Se depend heavily on a number of environmental parameters: the total Se content in the studied soil or water, the species involved in Se biomethylation, the prevailing temperature [276], the environment’s carbon content [17,262], the soil water content [276], and other parameters [277,286].

Furthermore, flux measurements do not necessarily reflect the amount of methylated species that has been produced in a certain environment. Methylated Se compounds are highly soluble and the concentrations in the aqueous-phase usually exceed those in the gaseous phase (as predicted by their Henry’s law constants [287]. Because the dissolved volatile Se species are also prone to (photo-) chemical or biological degradation and sorption to particles prior to volatilization [40,91,288,289], flux measurements may consistently underestimate biomethylation.

The biomethylation and volatilization of Se may critically affect the mobility and bioavailability of Se in the environment. Relative to the transport speeds in the vadose and saturated zones, atmospheric transport is comparatively fast. As a result, the transport of volatile species, including methylated and oxidized forms in the atmosphere, could be a major mechanism for redistributing Se at local to global scales.

## 8. Concluding Remarks

In this review we have addressed sources and pathways of Se across the soil-plant-atmosphere interfaces. Sources and sinks of Se, in addition to the speciation of Se along these pathways are all of key importance in determining Se content in soils and plants and thus in terrestrial food chains. The research presented in this review has been instrumental in providing an understanding of these sources and sinks, as well as the factors controlling plant uptake and losses, allowing us to evaluate the Se status of agricultural systems. Furthermore, understanding the mobility of Se and its interactions across system borders continues to be an important research activity since the environmental distribution of Se in the environment at different spatial and time scales is likely to be affected by environmental and climatic changes. Knowledge of the factors controlling distributions of Se in the environment will allow for current and future predictions of these distributions, which will be essential in the prevention of Se-related health hazards in the future.
